# Acetanilid (or Antifebrin)1Read before the semiannual meeting of the Pennsylvania State Veterinary Medical Association, October, 1896.

**Published:** 1897-02

**Authors:** F. S. Allen

**Affiliations:** Philadelphia


### ACETANILID (OR ANTIFEBRIN).[1]

By F. S. Allen, M.D., D.V.S.,
PHILADELPHIA.

(Concluded from page 12.)

In the winter of 1892 and 1893 I collected a few notes of cases treated by myself with acetanilid. While they are not as full and complete as I wish they were, they will help to illustrate its action, etc.

Case I.—Temperature 107.4°, falls in less than twenty-four hours to 102°, or 5° fall.

Case II.—Temperature 104°, falls in twenty-four hours to 100.6°, or 3.4°.

Case III.—Temperature 107°, falls in six hours to 105.6°, or fall = 1.4°; falls in twenty-four hours to 104° = fall 3° first day; 102.4° = fall 1.6° second day; 100.6° = 1.8° third day.

Case VI.—Temperature 105°, falls in twenty-four hours to 100.4° = fall 4.6° first day.

Case VII.—Temperature 104.2°, falls in twenty-four hours to 100.2° = fall 4° first day.

Case VIII.—Temperature 106.4°, falls in twenty-four hours to 104° = fall 2.4° first day; 101.6° = 2.4° second day; 100° = 1° third day.

Case IX.—Temperature 103°, falls in twenty-four hours to 100° = fall 3° first day.

Case X.—Temperature 105°, falls in twenty-four hours to 103° = fall 2° first day; 100° = fall 3° second day.

Case XI.—Temperature 105°, falls in five hours to 104.4° = fall $\frac{3}{5}$°; falls in twenty-four hours to 102.6° = fall 2.4° first day; became normal on sixth day.

Case V.—Temperature 104°, rose to 105° in five hours; rise 1°. It then began to fall to 104.6° = fall $\frac{2}{5}$° first day. It fell 2.8° on the fourth day and 1.8° on the fifth day. Normal on sixth day.

Case XII.—Temperature 106.2° at night. Temperature in morning 105.4°, fall of $\frac{3}{5}$° in twelve hours. Second day 104.2°, fall of 1.2°. Third day 104.8°, rise of $\frac{3}{5}$°. Fourth day 103.2°,

[1] Read before the semiannual meeting of the Pennsylvania State Veterinary Medical Association, October, 1896.

fall of 1.6°. It then began to fall gradually for six days. Acetanilid was renewed when it began to rise, continuing for two days, when it again fell gradually for two days and again rose till it reached 103.8°, then fell to nearly the normal, and recovery took place.

CASE XIII.—Typhoid. Bowels affected. Temperature 106.8° at night, in twelve hours reduced to 102°, fall of 4.8°. On the second day the temperature reduced to 100.8°, fall of 1.2°. The temperature continued at about 101° for a number of days, acetanilid being removed at the end of twenty-four hours. Laxatives, stimulants, and alteratives were given in its place. Recovery took place.

CASE XIV.—Typhoid. Lungs affected. Temperature at noon 106.6°, at 6 P.M. 106°, at 8 P.M. 108°; same day. Acetanilid was given for one day, when it was removed. Ice-water baths were introduced three times a day, from one hour to one hour and a half (and at times only half an hour). Alcohol was given in drink; alteratives, stimulants, and slight laxatives were given. Recovery in twelve days.

CASE XV.—Typhoid. Temperature 107°, falls in twenty-four hours to 106° = 1° fall first day; 104.6° = 1.4° second day; 104.4° = $\frac{1}{5}$° third day; 105° = $\frac{3}{5}$° rise fourth day; 106° = 1° rise fifth day; 105° = 1° fall sixth day; 103° = 2° fall seventh day; 101° = 2° fall eighth day; 103° = 3° rise ninth day. Died.

CASE XVI.—Temperature 105°, no fall first day; 105°, no fall second day; 104°, fall third day of 1°; 103.4°, fall fourth day of $\frac{3}{5}$°; 103°, fall fifth day of $\frac{2}{5}$°; 102°, fall sixth day of 1°; 100.6°, fall seventh day of 1.4°. Case taken out of my hands. My medicine continued to the last day, stimulants, etc. The case died some three or four days after leaving my hands. My successor having stopped all medicine for nearly one day, I informed the owner that the animal would die as a result, etc. When I left the owner my successor called and informed him the animal would recover. While reporting the case the animal died. The doctor had walked the animal into the yard and exercised him. I attributed the cause to over-exertion, etc.

Most of the cases were those of influenza—many of them green horses. Acetanilid was used in drachm doses and in combination with the fl. ext. belladonna, tr. nux. vom., tr. cinchona and alcohol. This mixture was given every three or four hours. In some cases I continued this mixture for six days, where fever so indicated, and I felt it was doing no harm. At other times it was used only one day and followed with nitrate of potassium in drinking-water. In other cases ammonium chloride, pulv. nux vom., quinin. sulph., etc., were given in bolus. In some cases I found the pulse became soft in character; there were at times great depression and weakness, the number of pulse-beats falling below the normal, as was the case in Case I. In other cases the pulse became intermittent. This I noticed mostly in typhoid diseases. In what is called pleuro-pneumonia contagiosa, etc., I have used acetanilid in drachm doses every three or four hours for twelve days, and in a few cases even longer, without any bad results.

This drug should be used with care, however, to prevent any accident. I can recall two cases—one in my practice and one in a friend's practice. My friend had just left the stable after giving a very favorable prognosis that the animal would recover, and was doing nicely, when the animal dropped dead. In my case the horse was small; the drug had been given, as was my custom, night and day every three or four hours. The animal was doing well when last seen the day before, but the treatment was to continue till the next day. On calling the next morning, to my surprise I learned the animal had died during the night, and had been removed. I felt that the drug should have been discontinued the night before, and it is possible by so doing the animal would have lived. I find that there is a tendency to over-stimulation of the kidneys at times and later to a suppression of the urine; these cases, as a rule, terminate fatally.

I have made it a rule of late to use acetanilid for twelve to twenty-four hours in all doubtful cases having a high fever, and thus learn if indicated or not.

I believe in full doses, and think it should be given once in four hours if we wish to obtain the best results. Eisenhart claims that small, repeated doses are useless. He says it should be given in large, full doses and at one time, and if no fall in temperature occurs resort to some other treatment.

---

ONE drachm of creolin added to a colic drench specially designed to relieve the pain incidental to gastric disturbance where there is a great accumulation of gas in the anterior portion of the gastro-intestinal tract oftentimes gives the most prompt relief in counteracting the fermentation.

